# Supplementary material for: Predicting visual function by interpreting a neuronal wiring diagram
Source: Nature. 2024 Oct 2;634(8032):113–23. doi: 10.1038/s41586-024-07953-5 (PMC11446822; doi:10.1038/s41586-024-07953-5)
Supplement: Supplementary file 6 — CRF and ERF predictions for individual TmY4 and TmY9 cells. Analogous to Supplementary Data 3, but for TmY target types. Shown are the top four monosynaptic pathways, the strongest pathway passing through each of the top ten intermediary types (ranking from Extended Data Fig. 7), and the trisynaptic pathway Tm1–TmY–Dm3–TmY (see the section entitled Prediction of spatial normalization). [file 41586_2024_7953_MOESM6_ESM.zip › DataS4/TmY9qΓèÑ/720575940617812692.pdf]

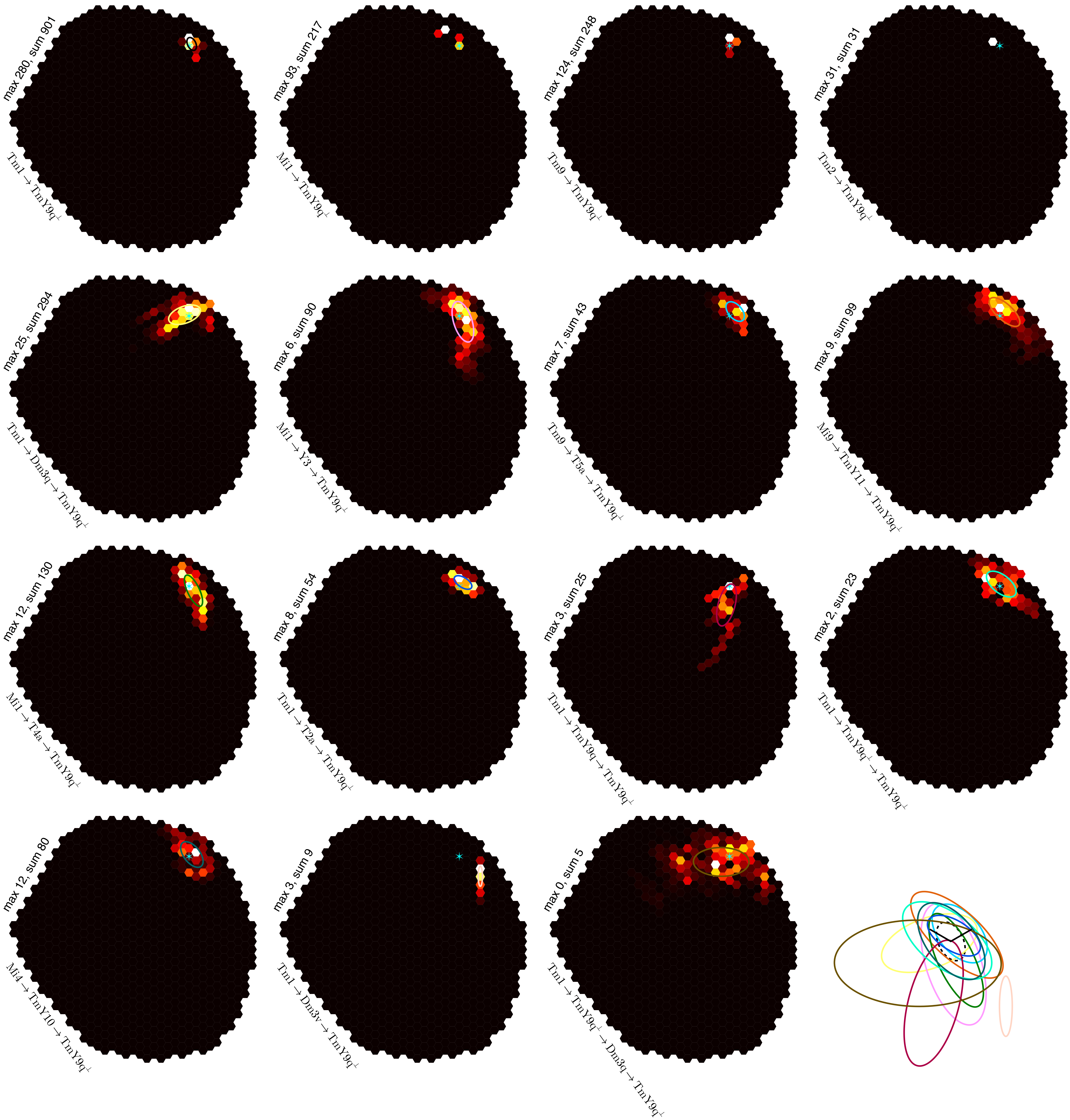

max 280, sum 901
Tm1 → TmY9q⊥
max 93, sum 217
Mi1 → TmY9q⊥
max 124, sum 248
Tm9 → TmY9q⊥
max 31, sum 31
Tm2 → TmY9q⊥
max 25, sum 294
Tm1 → Dm3q → TmY9q⊥
max 6, sum 90
Mi1 → Y3 → TmY9q⊥
max 7, sum 43
Tm9 → T5a → TmY9q⊥
max 9, sum 99
Mi9 → TmY11 → TmY9q⊥
max 12, sum 130
Mi1 → T4a → TmY9q⊥
max 8, sum 54
Tm1 → T2a → TmY9q⊥
max 3, sum 25
Tm1 → TmY9q → TmY9q⊥
max 2, sum 23
Tm1 → TmY9q⊥ → TmY9q⊥
max 12, sum 80
Mi4 → TmY10 → TmY9q⊥
max 3, sum 9
Tm1 → Dm3v → TmY9q⊥
max 0, sum 5
Tm1 → TmY9q⊥ → Dm3q → TmY9q⊥
